# The effect of radiation dose to the brain on early self-reported cognitive function in brain and head-and-neck cancer patients

**DOI:** 10.1016/j.ctro.2025.100929

**Published:** 2025-02-08

**Authors:** Femke Vaassen, David Hofstede, Catharina M.L. Zegers, Jeanette B. Dijkstra, Ann Hoeben, Monique H.M.E. Anten, Ruud M.A. Houben, Frank Hoebers, Inge Compter, Wouter van Elmpt, Daniëlle B.P. Eekers

**Affiliations:** a Department of Radiation Oncology (Maastro) GROW Research Institute for Oncology and Reproduction Maastricht University Medical Centre+ Maastricht the Netherlands; b Department of Medical Psychology Maastricht University Medical Center+ Maastricht the Netherlands; c Department of Medical Oncology GROW Research Institute for Oncology and Reproduction Maastricht University Medical Centre+ Maastricht the Netherlands; d Department of Neurology Maastricht University Medical Centre+ Maastricht the Netherlands

**Keywords:** Radiotherapy, Cognitive functioning, Cognitive decline, Patients-reported outcome measures, Quality of life, Radiation dose

## Abstract

**Purpose:**

Assess cognitive changes after radiotherapy (RT) in brain and head-and-neck (HN) cancer patients using patient-reported outcome measures (PROMs) and evaluate a dose–effect relationship for brain structures.

**Materials and methods:**

Primary brain and HN cancer patients treated with RT between 2012–2021 were included. Patient characteristics, clinical parameters, and PROMs at baseline and 1-year follow-up were collected. Cognitive functioning (CF) from the EORTC QLQ-C30, communication deficit (CD) from the QLQ-BN20, and one cognition-related questions from the EQ6D questionnaire were used, the latter two only for brain patients. Missing data were imputed and the four-point scale scores were transformed to a 100-point scale. Change in scores from baseline to 1-year were categorized into improvement/constant or deterioration. Organs-at-risk (OARs) were contoured either clinically or retrospectively using autocontouring and dose to the OARs were calculated.

**Results:**

A total of 110 brain and 356 HN cancer patients were included. Median age was 56 (brain) and 67.5 (HN) years. Baseline and 1-year CF was significantly lower for brain patients (p < 0.001). Univariate analysis for ΔCF showed that age at start RT ≤ 65 years, receiving chemotherapy, higher CF Baseline score, brain mean dose > 3 Gy, and multiple dose levels to left and right hippocampus were statistically associated with cognitive deterioration. Multivariate analysis for ΔCF identified age at RT ≤ 65 years, higher CF Baseline score, and brain mean dose > 3 Gy as significant predictors.

**Conclusion:**

This study identified risk factors for subjective cognitive decline and suggests that patients’ self-perceived cognitive deterioration may be related to age, CF baseline score and brain radiation dose above 3 Gy.

## Highlights

• Self-perceived cognitive decline after RT is important for brain and HN patients.
• There is a dose–effect relationship with cognitive decline for brain structures.
• Lower age, chemotherapy and higher baseline score are relevant parameters.
• Brain mean dose > 3 Gy is related to cognitive deterioration.

## Introduction

Radiation therapy (RT) often plays an important role in the multimodal treatment of brain and head-and-neck (HN) tumors. However, RT is also known for its association with short-term as well as long-term toxicity. One of the long-term complications most frequently affecting quality of life (QoL) is neurocognitive dysfunction [1], [2]. Over the past years, clinical trials have been including QoL and neurocognitive functioning in their outcome measures, but they are difficult to assess [3], [4], [5]. To evaluate early cognitive deterioration following RT for brain and HN tumors, extensive cognitive testing before and after treatment is optimal, but also demanding and time-consuming. Patient-reported outcomes measures (PROMs) are self-reported QoL questionnaires, which can be easily validated and implemented in clinical practice worldwide, possibly making them a good alternative to cognitive testing [6], [7].

The current literature presents contradictory results regarding the potential correlation between neurocognitive dysfunction and RT. Although several studies have failed to demonstrate a significant correlation between neurocognitive dysfunction and RT [8], [9], other studies did report neurocognitive impairments after RT, including declines in memory or attentional function [5], [10], [11], [12], [13]. It is well-established that both the radiation dose and the volume of irradiated healthy brain tissue are critical factors influencing neurocognitive (dys)function [14], [15].

Specific brain substructures are of importance with respect to irradiation and neurocognition. A significant association between radiation dose to the cerebellum and substructures and neurocognitive functioning over time has been shown [16], [17], [18]. Next to the cerebellum, the hippocampus is also a well-known organ-at-risk (OAR). Several studies have evaluated the benefits of performing hippocampal-sparing RT, demonstrating associations between hippocampal dose and cognitive functioning [19], [20], [21], [22], [23], [24], [25]. However, other studies observed preservation of cognitive functioning after RT for their patient cohort and no correlation with hippocampal doses [26].

PROMs have already been used to evaluate self-reported cognitive functioning in literature, both in brain and in HN patient groups. Possible association between high radiation doses and self-reported cognitive function were shown, but require validation [7], [27], [28].

As such, this study aimed to determine the changes in cognitive performance at 1-year after RT in patients with primary brain and HN cancer, measuring subjective cognitive function using PROMs. In addition, the dose–effect relationship for several brain structures, including the anterior and posterior cerebellum in relationship to cognitive changes was evaluated.

## Materials and methods

### Study population

Brain and HN cancer patients treated with RT between 2012–2021 at Maastro Clinic were included in this study. The inclusion criteria were primary brain tumors, HN tumors, and age ≥ 18 years. The total treatment strategy involved radiotherapy in a primary or post-operative setting and/or (concurrent) chemoradiation. Patients were included in a standard follow-up program, containing prospective longitudinal assessment of outcome using PROMS and toxicity using CTC-criteria (clinicaltrial.gov ID: NCT01985984). Patients with previous cranial/HN cancer irradiation or hypo-fractionated stereotactic irradiation were excluded. The Institutional Review Board approved this retrospective study with project number P0461.

### Organ-at-risk delineation

All patients underwent pre-treatment (planning) computed tomography (CT)-brain with a slice thickness of 3 (HN) or 1 mm (brain) according to protocol (SOMATOM Drive or Confidence; Siemens, Erlangen, Germany). Brain patients also had a 3D T1-weighted neuronavigation post-gadolinium contrast magnetic resonance imaging (MRI) scan with a 1 mm slice thickness (Ingenia CX or Achieva; Philips, Best, The Netherlands). HN cancer patients received MRI imaging as indicated (nasopharyngeal or oropharyngeal cancer). CT scans were registered to the 3D T1-weighted MRI scan when available.

For the brain cancer patients, the brain, brainstem, and left and right hippocampus were delineated in clinical routine on registered CT-MRI scans using the EPTN-guidelines [29], [30], [31]. For the supratentorial brain and whole cerebellum, a CT-based deep-learning autosegmentation model (DLCExpert, Mirada Medical Ltd., Oxford, UK) was used. For the anterior and posterior cerebellum, a research version of an MRI-based deep-learning autosegmentation model (DLCExpert, Mirada Medical Ltd., Oxford, UK) was used for the brain patients, and an atlas-based model (Embrace, Mirada Medical Ltd., Oxford, UK) trained on neuro-oncological patients was used for the HN patients [32].

Radiation treatment plans (RTPLAN) and doses (RTDOSE) were collected. For the patients with multiple treatment plans, a weighted average of RTDOSE files was calculated. All post-processing was performed in Matlab R2021a (The MathWorks Inc., Natick, MA, USA).

### Dose-volume parameters

For each OAR, mean doses were calculated. For both hippocampi, the dose to 40 % of the volume was calculated as well. Dose values were converted to biologically equivalent doses in 2 Gy fractions (EQD2) assuming an α/β ratio of 2 as mentioned by Lambrecht et al. to account for different fractionation schemes [33]. Dose-volume parameters over all patients are reported as median (IQR) in Gy and dichotomized. Limits for dichotomization to consider low dose regions were selected in two ways: 1) the median DVH values for the total patient group (see Supplementary Material Table S4), and 2) the DVH value in a range of 1–5 Gy which had the highest statistical significance in the univariate analysis.

### Patient-reported cognitive function

Neurocognitive functioning was assessed with the European Organization for Research and Treatment of Cancer Quality-of-Life Instrument (EORTC QLQ-C30) [34]. Two four-point scale questions relating to cognition (Q20: “Have you had difficulty in concentrating on things, like reading a newspaper or watching television?” and Q25: “Have you had difficulty remembering things?”) were included in this study. Scores were combined and transformed to a 100-point Cognitive Functioning (CF) scale, where a higher score means a better functioning [35].

Next to the QLQ-C30 questionnaire, the brain patients also received the EORTC QLQ-BN20 questionnaire [36] and the Six-Dimensional EuroQol instrument (EQ6D) [37]. Three four-point scale questions relating to communication deficit (Q11, Q12, and Q13) from the EORTC QLQ-BN20 were included in this study. Scores were combined and transformed to a 100-point Communication Deficit (CD) scale, where a lower score means a better functioning [35]. One five-point scale question about cognitive functioning from the EQ6D was included, where a lower score means a better functioning.

PROMs data were collected at baseline and 1-year follow-up after RT. Per patient, the change in Cognitive Functioning score (ΔCF), Communication Deficit score (ΔCD) and EQ6D score (ΔEQ6D) were determined from baseline to 1-year follow-up and assigned to one of two subgroups (group 1: improvement/constant score, group 2: deterioration). Dichotomization was performed per functioning scale to create a binary classification. ΔCF and ΔCD of at least 7 was considered a relevant change [38], [39]. For ΔED6D, a change of at least 1 was considered relevant.

### Statistical analysis

Statistical analysis was performed using IBM SPSS Statistics (Version 29). A p < 0.05 was considered statistically significant. When missing data was observed for one question, substitution of the total scale to be based on one answered question was performed. When missing data of all questions at baseline or 1-year was observed, imputation of this data was performed in SPSS using the multiple imputation function (5 imputations and using both variables as predictors). General compliance to PROMs is approximately 60 % at baseline and 75 % at one-year in our institution.

Univariate logistic regression was performed for ΔCF, ΔCD and ΔEQ6D, to determine the associations with patient characteristics as well as dose-volume parameters. Odds ratios were calculated. For the parameter age the median age at the start of RT of total patient group was considered. The dose-volume parameters were dichotomized based on specific dose thresholds. Multivariate logistic regression was performed to include all possible associated parameters together. Variables with a high collinearity were excluded. First a model was fitted using only patient characteristics: primary disease site, age at RT ≤ 65 years, female gender, chemotherapy, surgery and baseline score. Secondly, a model was fitted using only dose-volume measures. Patient characteristics and dose-volume measures were included in combined models.

## Results

A total of 110 brain and 356 HN cancer patients were included. Of the brain patients, 35 (32 %) patients had a benign brain tumor, 36 (33 %) patients had a glioma WHO grade II or III tumor (astrocytoma, oligodendroglioma, or ependymoma), 34 (31 %) patients had a glioma WHO grade IV tumor, and the remaining 5 (4 %) patients were classified as various other malignant tumor types.

Median age at the start of RT was 56 years for brain cancer patients and 67.5 years for HN cancer patients. For the total patient group, median age at start RT was 65 years. Of the brain patients, 67 (67 %) received chemotherapy as part of their treatment, of whom 53 (79 %) received temozolomide (TMZ) and 14 (21 %) received PCV (combination of procarbazine, lomustine (CCNU) and vincristine). Of the HN patients, 95 (26.7 %) received systemic cancer therapy, of whom 76 patients (80 %) received cisplatin, 2 (2 %) carboplatin, and 17 (18 %) cetuximab. Hereafter, this will all be referred to as chemotherapy for simplicity.

Of the brain patients, 73 (66.4 %) received photon therapy, 4 (3.6 %) received only proton therapy, and 33 (30 %) received a combination of proton and photon therapy. The use of this combined therapy was necessary due to the downtime of the proton therapy machine. Some patients also received plan adaptation, which could be due to e.g. anatomical variations or tumor shrinkage. All statistics on the patient sample are presented in Table 1. Information on the fractionation schemes and available PROMs and dosimetric data can be found in Supplementary Material Table S1 and S2.

**Table 1:** Baseline patient characteristics and treatment information. Bold values represent statistically significant different values (Chi^2^ or Mann-Whitney *U* test) between brain and head-and-neck patient groups. HN = head-and-neck. RT = radiotherapy.

**Variable**	**Brain patients (n = 110)**	**HN patients (n = 356)**	*p-value (Brain* vs*. HN patients)*
**Gender**			**<0.001**
Male	48 (43.6 %)	266 (74.7 %)	
Female	62 (56.4 %)	90 (25.3 %)	
**Age (in years)**			**<0.001**
Mean	55.3	67.0	
Median	56	67.5	
Range	21–90	21–93	
Age ≤ 65 years	83 (75.5 %)	149 (41.9 %)	
**Surgery**			**<0.001**
Yes	86 (78.2 %)	81 (22.8 %)	
No	24 (21.8 %)	275 (77.2 %)	
**Chemotherapy**			**<0.001**
Yes	67 (61 %)	95 (26.7 %)	
No	43 (39 %)	261 (73.3 %)	
**Modality of RT**			**<0.001**
Photon	73 (66.4 %)	350 (98.3 %)	
Proton	4 (3.6 %)	−	
Combined	33 (30 %)	6 (1.7 %)	
**Plan adaptation**			0.384
Yes	15 (13.6 %)	34 (9.6 %)	
No	95 (86.4 %)	322 (90.4 %)	

**Fig. 1:**
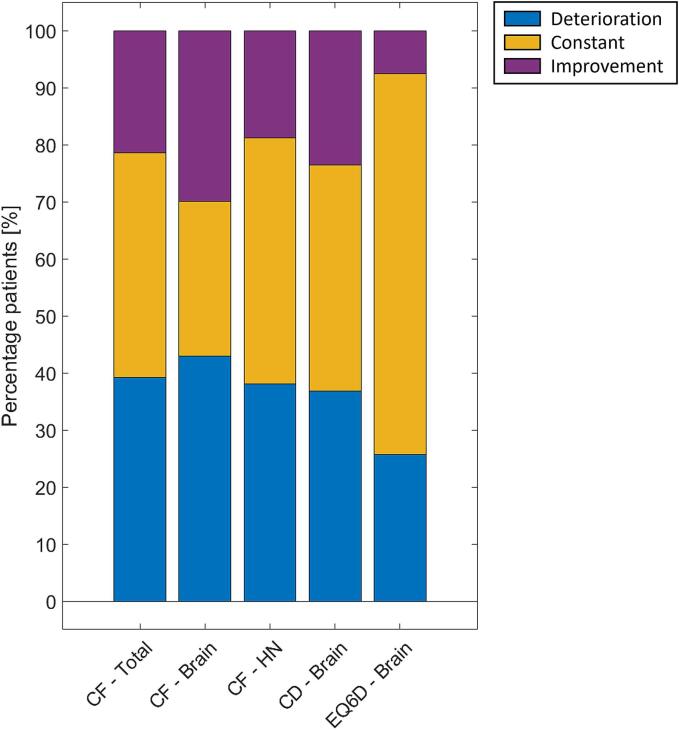
Percentage of patients with a deterioration, constant or improvement for CF (QLQ-C30), CD (QLQ-BN20), and EQ6D at 1-year after RT. Improvement and deterioration are defined to be a minimum change of 7 points in either direction for CF and CD, and a minimum change of 1 point for EQ6D. In the further analysis, constant and improvement scores are combined. HN = head-and-neck, CF = Cognitive Functioning, CD = Communication Deficit.

Imputation of missing data at baseline and 1-year was performed when at least one of the two timepoints was present, following the procedure as described in the Methods. This resulted in PROMs ΔCF data for 462 patients, ΔCD data for 106 patients, and ΔEQ6D data for 105 patients. All information about the PROMs scores is given in Supplementary Material Table S3. Distributions of the delta scores after imputation can be seen in Fig. 1. Deterioration in ΔCF occurred in a total of 181/462 (39 %) patients of whom 46/107 (43 %) were brain cancer patients and 135/355 (38 %) HN cancer patients. Deterioration in ΔCD occurred in 39/106 (36.8 %) brain cancer patients, and deterioration in ΔEQ6D occurred in 27/105 (25.7 %) brain cancer patients.

A statistically significant difference (p < 0.001) in mean dose to OARs between brain and HN cancer patients was found for all OARs except for the total cerebellum (median (IQR): 2.1(7.7) Gy vs. 2.0(5.0) Gy, p = 0.843). For brain cancer patients, supratentorial brain received the highest mean dose (8.2(13.3) Gy). For HN cancer patients, the posterior cerebellum received the highest mean dose (3.0(5.6) Gy). All dosimetric values are given in Supplementary Material Table S4 and Fig. S1.

### Univariate analyses

**Fig. 2:**
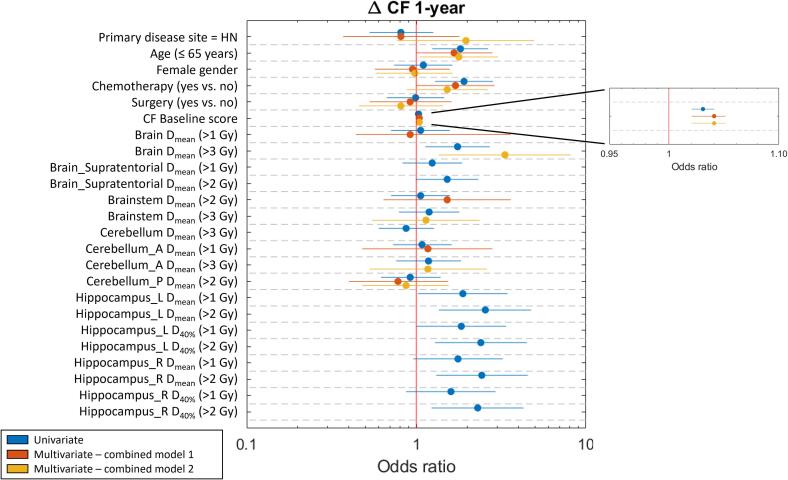
Odds ratios and 95 % confidence interval of the univariate and multivariate combined analyses performed for 1-year ΔCF. When the horizontal line is not overlapping with the red vertical line, the variable has a significant association. CF = Cognitive Functioning, HN = head-and-neck, Dmean = mean dose, D40% = dose to 40 % of the structure volume, Cerebellum_A = anterior cerebellum, Cerebellum_P = posterior cerebellum, Hippocampus_L = left hippocampus, Hippocampus_R = right hippocampus. Δ scores are defined in 2 groups (group 1: improvement or constant, group 2: deterioration). (For interpretation of the references to colour in this figure legend, the reader is referred to the web version of this article.)

Univariable logistic regression of patient characteristics as well as clinical variables and dose-volume parameters for ΔCF is presented in Fig. 2. Quantitative results are given in Supplementary Material Table S5 and S6. Considering ΔCF, age at RT ≤ 65, receiving chemotherapy as part of treatment, higher CF Baseline score (i.e. better functioning), brain mean dose > 3 Gy, left hippocampus mean dose > 1 Gy and > 2 Gy, right hippocampus mean dose > 2 Gy, left hippocampus D_40%_ >1Gy and > 2 Gy, and right hippocampus D_40%_ >2Gy were statistically positively associated with 1-year cognitive deterioration. Cross tabulations of a selection of these associations are shown in Table 2. Lower CD Baseline score (i.e. better functioning) was significantly associated with deterioration ΔCD. For ΔEQ6D, receiving chemotherapy, brain mean dose > 8 Gy, and supratentorial brain mean dose > 8 Gy were statistically positively associated with 1-year deterioration.

**Table 2:** Cross tabulations of a selection of the univariate analyses with significant associations for ΔCF. The numbers represent the absolute count of patients, and in brackets the percentage within the ΔCF group. RT = radiotherapy, CF = Cognitive Functioning, Hippocampus_L = left hippocampus, Hippocampus_R = right hippocampus, D_mean_ = mean dose, D_40%_ = dose to 40 % of the structure volume.

		**ΔCF**		
		**Improvement/** **constant**	**Deterioration**	**Total**
Age at start RT	≤65 years	123 (43.8 %)	106 (58.6 %)	229
	*>65 years*	158 (56.2 %)	75 (41.4 %)	233
	*Total*	281	181	462
*Chemo-* *therapy*	*No*	200 (71.2 %)	102 (56.4 %)	302
	*Yes*	81 (28.8 %)	79 (43.6 %)	160
	*Total*	281	181	462
*Brain D_mean_*	*≤3 Gy*	194 (77.3 %)	109 (66.1 %)	303
	*>3 Gy*	57 (22.7 %)	56 (33.9 %)	113
	*Total*	251	165	416
*Hippocampus_L D_mean_*	*≤2 Gy*	77 (70 %)	33 (47.8 %)	110
	*>2 Gy*	33 (30 %)	36 (52.2 %)	69
	*Total*	110	69	179
*Hippocampus_L D_40%_*	*≤2 Gy*	77 (70 %)	34 (49.3 %)	111
	*>2 Gy*	33 (30 %)	35 (50.7 %)	68
	*Total*	110	69	179
*Hippocampus_R D_mean_*	*≤2 Gy*	78 (70.3 %)	34 (49.3 %)	112
	*>2 Gy*	33 (29.7 %)	35 (50.7 %)	68
	*Total*	111	69	180
*Hippocampus_R D_40%_*	*≤2 Gy*	78 (70.3 %)	35 (50.7 %)	113
	*>2 Gy*	33 (29.7 %)	34 (49.3 %)	67
	*Total*	111	69	180

Specific types of chemotherapy were also tested for significance (see Supplementary Material Table S7). For the brain patients, TMZ showed a statistically significant correlation with ΔCF and ΔEQ6D deterioration. For the HN patients, cisplatin showed a statistically significant correlation with ΔCF.

### Multivariate analyses

Multivariate logistic regression was performed for ΔCF, ΔCD and ΔEQ6D. Results for ΔCF can be found in Table 3 and Fig. 2, and for ΔCD and ΔEQ6D in Supplementary Material Table S8. In the patient characteristics model, age at RT ≤ 65 years (OR = 1.73, 95 % CI = 1.11–2.70, p = 0.016), receiving chemotherapy (OR = 1.59, 95 % CI = 1.00–2.52, p = 0.049), and higher CF Baseline score, i.e. better functioning (OR = 1.03, 95 % CI = 1.02–1.05, p < 0.001) were significantly associated with ΔCF deterioration. Receiving chemotherapy (OR = 4.31, 95 % CI = 1.31–14.14, p = 0.016) was significantly associated with ΔEQ6D deterioration. Lower CD Baseline score, i.e. better functioning (OR = 0.95, 95 % CI = 0.92–0.98, p = 0.001) was significantly associated with ΔCD deterioration.

**Table 3:** Multivariate logistic regression analysis of patient characteristics, clinical variables and dose-volume parameters for 1-year ΔCF. Δ scores are defined in 2 groups (group 1: improvement or constant, group 2: deterioration). CF = Cognitive Functioning, OR = Odds Ratio, CI = Confidence Interval, HN = head-and-neck, D_mean_ = mean dose, Cerebellum_A = anterior cerebellum, Cerebellum_P = posterior cerebellum. Bold values represent statistically significant associations.

	**ΔCF 1-year**	
	**OR (95 % CI)**	**p**
**Patient characteristics model**	n = 462	
Primary disease site = HN	0.76 (0.42 to 1.36)	0.350
Age at start RT ≤ 65 years	1.73 (1.11 to 2.70)	**0.016**
Gender = female	1.08 (0.70 to 1.69)	0.722
Chemotherapy = yes	1.59 (1.00 to 2.52)	**0.049**
Surgery = yes	0.91 (0.56 to 1.48)	0.688
CF Baseline score	1.03 (1.02 to 1.05)	**<0.001**
**Dose-volume measures model 1 (median DVH values)**	n = 346	
Brain D_mean_ (> 1 Gy vs. ≤ 1 Gy)	1.00 (0.58 to 1.73)	0.992
Brainstem D_mean_ (> 2 Gy vs. ≤ 2 Gy)	1.12 (0.49 to 2.56)	0.786
Cerebellum_A D_mean_ (> 1 Gy vs. ≤ 1 Gy)	1.29 (0.55 to 3.02)	0.554
Cerebellum_P D_mean_ (> 2 Gy vs. ≤ 2 Gy)	0.80 (0.44 to 1.46)	0.471
**Dose-volume measures model 2 (most significant DVH values)**	n = 346	
Brain D_mean_ (> 3 Gy vs. ≤ 3 Gy)	2.03 (1.14 to 3.62)	**0.016**
Brainstem D_mean_ (> 3 Gy vs. ≤ 3 Gy)	0.99 (0.50 to 1.94)	0.972
Cerebellum_A D_mean_ (> 3 Gy vs. ≤ 3 Gy)	0.92 (0.44 to 1.93)	0.833
Cerebellum_P D_mean_ (> 2 Gy vs. ≤ 2 Gy)	1.07 (0.63 to 1.79)	0.812
**Combined model 1 (median DVH values)**	n = 346	
Primary disease site = HN	0.81 (0.37 to 1.79)	0.607
Age at start RT ≤ 65 years	1.67 (0.99 to 2.81)	0.056
Gender = female	0.95 (0.57 to 1.57)	0.844
Chemotherapy = yes	1.70 (0.99 to 2.89)	0.052
Surgery = yes	0.92 (0.53 to 1.61)	0.779
CF Baseline score	1.04 (1.02 to 1.05)	**<0.001**
Brain D_mean_ (> 1 Gy vs. ≤ 1 Gy)	0.92 (0.44 to 3.60)	0.816
Brainstem D_mean_ (> 2 Gy vs. ≤ 2 Gy)	1.52 (0.64 to 3.60)	0.340
Cerebellum_A D_mean_ (> 1 Gy vs. ≤ 1 Gy)	1.17 (0.48 to 2.81)	0.733
Cerebellum_P D_mean_ (> 2 Gy vs. ≤ 2 Gy)	0.78 (0.40 to 1.54)	0.477
**Combined model 2 (most significant DVH values)**	n = 346	
Primary disease site = HN	1.96 (0.77 to 4.96)	0.157
Age at start RT ≤ 65 years	1.78 (1.05 to 3.03)	**0.033**
Gender = female	0.98 (0.58 to 1.64)	0.930
Chemotherapy = yes	1.52 (0.88 to 2.64)	0.137
Surgery = yes	0.81 (0.46 to 1.44)	0.478
CF Baseline score	1.04 (1.02 to 1.05)	**<0.001**
Brain D_mean_ (> 3 Gy vs. ≤ 3 Gy)	3.33 (1.36 to 8.14)	**0.008**
Brainstem D_mean_ (> 3 Gy vs. ≤ 3 Gy)	1.14 (0.55 to 2.36)	0.734
Cerebellum_A D_mean_ (> 3 Gy vs. ≤ 3 Gy)	1.17 (0.53 to 2.60)	0.701
Cerebellum_P D_mean_ (> 2 Gy vs. ≤ 2 Gy)	0.87 (0.48 to 1.55)	0.623

In the dose-volume models, the supratentorial brain was excluded due to high collinearity with the brain (−0.843), and the whole cerebellum was excluded due to high collinearity with the posterior cerebellum (−0.837). Left and right hippocampus dose-volume measures were excluded in multivariate analyses because the number of patients with dose information for these structures was low (179 and 180, respectively). For HN patients, the hippocampi are only relevant when the tumor is located cranially, limiting the number of delineations present. Brain mean dose > 3 Gy was significantly associated with ΔCF deterioration in the model using the most significant DVH values (OR = 2.03, 95 % CI = 1.14–3.62, p = 0.016). For ΔCD and ΔEQ6D, no DVH values were statistically significant.

For ΔCF, in the combined model using the median DVH values, only higher CF Baseline score (OR = 1.04, 95 % CI = 1.02–1.05, p < 0.001) showed a significant association. Age at RT ≤ 65 years and receiving chemotherapy were close to significance. In the model using the most significant DVH values on univariate analysis however, age at RT ≤ 65 years (OR = 1.78, 95 % CI = 1.05–3.03, p = 0.033), higher CF Baseline score (OR = 1.04, 95 % CI = 1.02–1.05, p < 0.001), and brain mean dose > 3 Gy (OR = 3.33 95 % CI = 1.36–8.14, p = 0.008) showed a significant association. For ΔEQ6D, no variables were statistically significant in both combined models, and for ΔCD, only lower CD Baseline score was statistically significant in both combined models. Regression weights of these multivariate models are presented in Supplementary Material Table S9 and S10.

## Discussion

Neurocognitive decline is a well-known side-effect of radiotherapy for patients treated for tumors in the brain region. To evaluate this neurocognitive (dys)function before and after treatment, PROMs questionnaires have been used [7]. In this study, we aimed to determine the changes in self-reported cognitive functioning at 1-year after RT with the aid of PROMs. Additionally, we studied the dose–effect relationship for several brain structures with respect to cognitive changes.

Our results highlight the relevance of specific dose regions in the brain in relation to cognitive deterioration. We found that brain mean dose > 3 Gy and several dose levels to the left and right hippocampus were positively associated with 1-year cognitive deterioration in univariate analyses. Due to the limited dosimetric information available for the hippocampi, this variable was excluded in the combined model, leaving the mean brain dose > 3 Gy as the most relevant dosimetric variable. In a combined model including patient characteristics and the most significant DVH values, this correlation was still statistically significant (p = 0.008). These results indicate that a dose to the total brain above 3 Gy is relevant in cognitive functioning.

The univariate analyses showed that age at RT ≤ 65, receiving chemotherapy as part of the treatment, and higher baseline score were positively associated with 1-year cognitive deterioration. Results in literature about the influence of age on neurocognitive decline are varying [15]. Some studies are in line with our results reporting more cognitive deterioration for lower age, but other studies show the opposite [40]. There might be a difference between objective and subjective cognitive deterioration considering age-dependence, as younger patients might perceive cognitive deterioration worse compared with older patients. Here, brain patients had a lower age compared to HN patients (median 56 vs. 67.5 years), which might also influence the perception of cognitive deterioration.

Next to a correlation with age, receiving chemotherapy as part of treatment appeared relevant. This is in agreement with some studies that also reported a correlation with chemotherapy [41], [42]. We found a significant correlation with chemotherapy type TMZ for brain patients, and with cisplatin for HN patients. In contrast, other studies reported no correlation of chemotherapy with cognitive decline [43], [44], [45]. In our multivariate combined models for ΔCF, chemotherapy was no longer significantly associated compared to the univariate analysis, indicating that receiving chemotherapy as part of the treatment regime is relevant for cognitive deterioration, but more research would be recommended to validate these findings.

Patients who self-reported a higher cognitive functioning at baseline (i.e. higher CF Baseline score or lower CD Baseline score) were shown to be at higher risk of neurocognitive deterioration at 1-year. This variable remained significant in the multivariate models, indicating that this is a relevant factor for predicting neurocognitive decline, which has been found in other studies as well [46], [47]. A potential explanation for this correlation could be that patients with a higher baseline cognitive functioning are more aware of their cognitive decline and thus report lower scores after RT.

Mean dose to the cerebellum was not significantly associated with cognitive deterioration in this study. Other studies report varying results about a potential correlation between cognitive decline and radiation dose to (part of) the cerebellum [7], [16], [18]. We can conclude that we did not find a correlation between mean dose to the cerebellum (or any subparts of the cerebellum) and patient-reported cognitive deterioration. It is still recommended to keep reporting the dose to the cerebellum, because this study only included subjective measures. Tumor location will influence the dose to the cerebellum, and also baseline cognitive function [48].

A limitation of this study is the sample size, especially for CD and EQ6D as only brain patients received these specific questionnaires. The number of patients with hippocampi delineated was also limited, especially for HN patients. This limits the interpretation of the multivariate analyses. Compliance to PROMs questionnaires is approximately 60 % at baseline and 75 % at one-year, which could introduce participation bias. Imputation of missing PROMs data both at baseline and 1-year was performed to increase the sample size as much as possible, where we assumed that the data was missing-at-random. Including more patients could result in stronger correlations, so further validation of the results is recommended. Additionally, internal and external validation would be recommended to be able to generalize the results between institutions. Another limitation of this study is that we used a follow-up period of only 1-year after RT. Cognitive deterioration is known to be a long-term toxicity, but it has been shown that cognitive deterioration can be permanent at 1-year after RT [49]. Further research should be focused on longer follow-up to validate the findings presented in this study.

In conclusion, this study identified in a cohort of patients with head-and-neck and brain cancer that patient-reported cognitive deterioration is influenced by an age below 65 years, receiving chemotherapy as part of the treatment, baseline cognitive functioning score, and a higher mean dose to the brain. Higher dose to the cerebellum was not associated with cognitive deterioration.

## Patient consent statement

Not applicable.

## CRediT authorship contribution statement

**Femke Vaassen:** Conceptualization, Data curation, Methodology, Visualization, Writing – original draft. **David Hofstede:** Data curation, Methodology, Writing – review & editing. **Catharina M.L. Zegers:** Supervision, Visualization, Writing – review & editing. **Jeanette B. Dijkstra:** Writing – review & editing. **Ann Hoeben:** Writing – review & editing. **Monique H.M.E. Anten:** Writing – review & editing. **Ruud M.A. Houben:** Methodology, Writing – review & editing. **Frank Hoebers:** Writing – review & editing. **Inge Compter:** Writing – review & editing. **Wouter van Elmpt:** Writing – review & editing. **Daniëlle B.P. Eekers:** Supervision, Visualization, Writing – review & editing.

## Declaration of competing interest

The authors declare that they have no known competing financial interests or personal relationships that could have appeared to influence the work reported in this paper.
