# Supplementary material for: The effect of radiation dose to the brain on early self-reported cognitive function in brain and head-and-neck cancer patients
Source: Clin Transl Radiat Oncol. 2025 Feb 8;52:100929. doi: 10.1016/j.ctro.2025.100929 (PMC11869991; doi:10.1016/j.ctro.2025.100929)
Supplement: Supplementary Data 1 [file mmc1.pdf]

SUPPLEMENTARY MATERIAL

The effect of radiation dose to the brain on early self-reported cognitive function in brain and head-and-neck cancer patients

Table S1 – Information on the fractionation schemes of the patients included in the study. HN = head-and-neck.

**Brain patients (n=110)**		**HN patients (n=355)**	
Fractionation scheme	*Number of patients (%)*	*Fractionation scheme*	*Number of patients (%)*
33 x 1.8 Gy (59.4 Gy)	37 (33.6%)	35 x 2 Gy (70 Gy)	140 (39.4%)
28 x 1.8 Gy (50.4 Gy)	30 (27.3%)	34 x 2 Gy (68 Gy)	106 (29.9%
30 x 1.8 Gy (54 Gy)	16 (14.5%)	33 x 2 Gy (66 Gy)	68 (19.2%)
30 x 2 Gy (60 Gy)	15 (13.6%)	25 x 2.4 Gy (60 Gy)	26 (7.3%)
15 x 2.67 Gy (40.05 Gy)	6 (5.5%)	28 x 2 Gy (56 Gy)	11 (3.1%)
20 x 2 Gy (40 Gy)	1 (0.9%)	20 x 2.4 Gy (48 Gy)	2 (0.6%)
33 x 2 Gy (66 Gy)	1 (0.9%)	30 x 2 Gy (60 Gy)	1 (0.3%)
26 x 1.8 Gy (46.8 Gy)	1 (0.9%)	12 x 2 Gy (24 Gy)	1 (0.3%)
25 x 1.8 Gy (45 Gy)	1 (0.9%)		
10 x 3.5 Gy (35 Gy)	1 (0.9%)		
29 x 1.8 Gy (52.2 Gy)	1 (0.9%)		

Table S2 – The number of patients for which the PROMs and dosimetric data was available. HN = head-and-neck. RT = radiotherapy, CF = Cognitive Functioning, CD = Communication Deficit.

**Variable**	**Brain patients (n=110)**	**HN patients (n=356)**
**PROMs CF available**		
At baseline	107	354
At 1-year	86	226
**PROMs CD available**		
At baseline	106	-
At 1-year	84	-
**PROMs EQ6D available**		
At baseline	103	-
At 1-year	82	-
**Dosimetric data available**		
Brain D_{mean}	108	312
Brain_Supratentorial D_{mean}	108	329
Brainstem D_{mean}	102	298
Cerebellum D_{mean}	106	355
Cerebellum_A D_{mean}	100	300
Cerebellum_P D_{mean}	97	296
Hippocampus_L D_{mean}	105	77
Hippocampus_L $D_{40\%}$	105	77
Hippocampus_R D_{mean}	106	77
Hippocampus_R $D_{40\%}$	106	77

Table S3 – All PROMS scores. Values are reported as median (IQR), for all patients together, as well as for the brain and HN patients only. Bold values represent statistically significant different values (Mann-Whitney U-test, p<0.05) between brain and HN patients. HN = head-and-neck, CF = Cognitive Functioning, CD = Communication Deficit.

	**PROMs scores**		
	All patients	*Brain patients*	*HN patients*
CF Baseline	100 (33.3)	**83.3 (50)**	**100 (16.7)**
CF 1-year	83.3 (32.9)	**71.0 (41.3)**	**83.3 (23.6)**
ΔCF 1-year	0 (16.7)	0 (33.3)	0 (16.7)
CD Baseline		11.1 (22.2)	
CD 1-year		15.7 (28.3)	
ΔCD 1-year		0 (14.7)	
EQ-6D Baseline		2 (1)	
EQ-6D 1-year		2 (2)	
ΔEQ6D 1-year		0 (1)	

Table S4 – Overview of dosimetric values for the selected structures. Reported are the number of patients for which a delineation of the structure was present. Values are reported as median (IQR), for all patients together, as well as for the brain and HN patients only. Bold values represent statistically significant different values (Mann-Whitney U-test) between brain and head-and-neck patient groups. HN = head-and-neck.

	**Dosimetric value [Gy]**			
	All patients	*Brain patients only*	*HN patients only*	*p-value (brain vs. HN)*
Brain D_{mean}	0.8 (2.9)	7.7 (11.4)	0.6 (0.8)	**<0.001**
Brain_Supratentorial D_{mean}	0.4 (2.1)	8.2 (13.3)	0.3 (0.3)	**<0.001**
Brainstem D_{mean}	2.0 (5.9)	8.0 (15.9)	1.6 (3.3)	**<0.001**
Cerebellum D_{mean}	2.0 (5.5)	2.1 (7.7)	2.0 (5.0)	0.843
Cerebellum_A D_{mean}	0.9 (3.5)	6.2 (19.3)	0.8 (1.4)	**<0.001**
Cerebellum_P D_{mean}	2.5 (5.6)	1.5 (5.7)	3.0 (5.6)	**0.002**
Hippocampus_L D_{mean}	0.9 (3.6)	2.4 (11.7)	0.5 (0.5)	**<0.001**
Hippocampus_L $D_{40\%}$	0.8 (3.4)	2.3 (12.2)	0.5 (0.6)	**<0.001**
Hippocampus_R D_{mean}	0.8 (4.0)	3.2 (7.2)	0.4 (0.4)	**<0.001**
Hippocampus_R $D_{40\%}$	0.7 (3.7)	3.1 (6.5)	0.5 (0.4)	**<0.001**

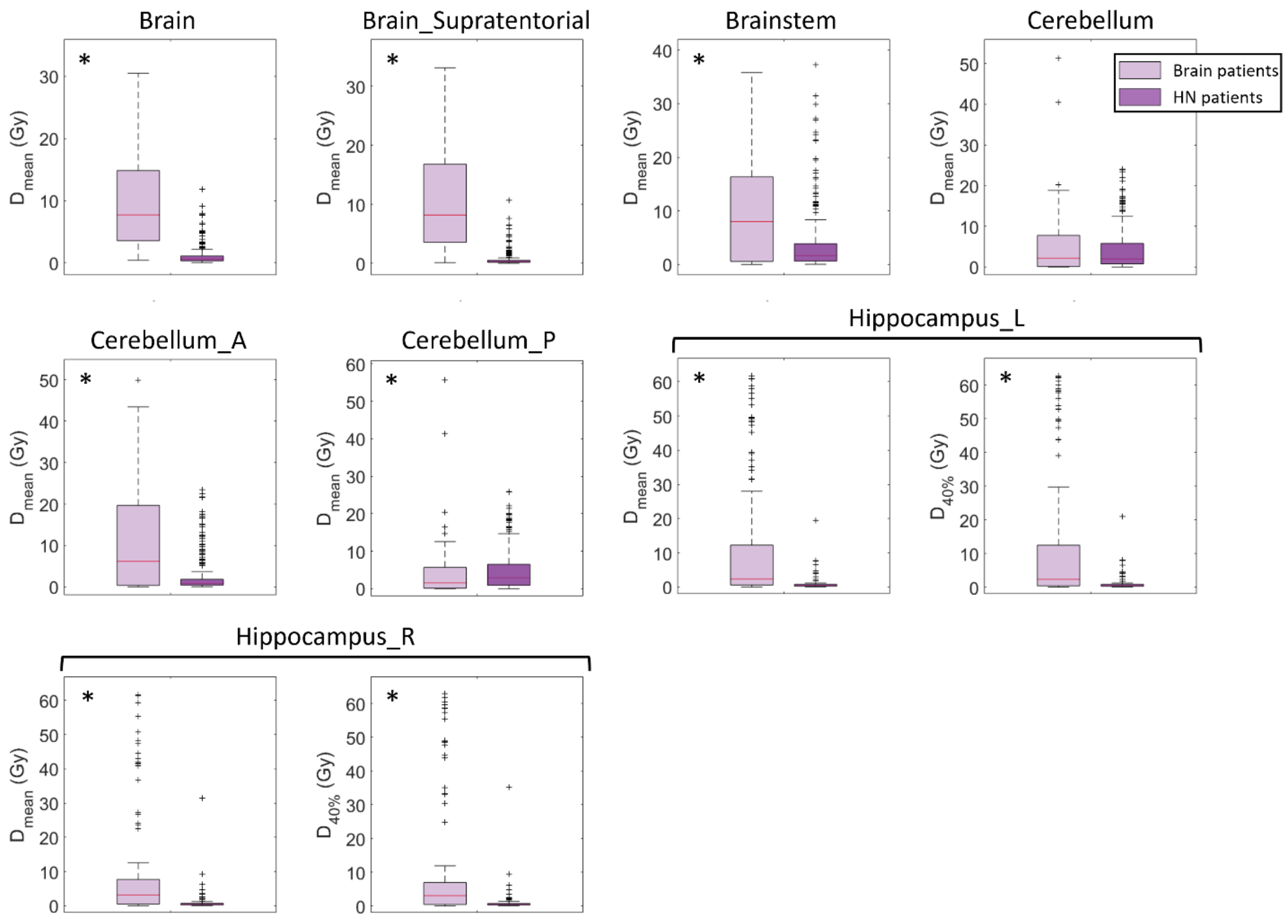

*Figure S1 – Dosimetric values for the selected structures comparing brain and head-and-neck patient groups. * = statistically significant different values (Mann-Whitney U-test) between brain and head-and-neck patient groups. HN = head-and-neck, D_{mean} = mean dose, $D_{40\%}$ = dose to 40% of the structure volume, Cerebellum_A = anterior cerebellum, Cerebellum_P = posterior cerebellum, Hippocampus_L = left hippocampus, Hippocampus_R = right hippocampus.*

Table S5 – Univariate logistic regression analysis of the patient characteristics, clinical variables and dose-volume parameters for 1-year ΔCF. The number of patients per variable included are given. Δ scores are defined in 2 groups (group 1: improvement or constant, group 2: deterioration). For the dosimetric variables, the limits for dichotomization based on the median DVH values for the total patient group (see Table S4) are represented with a grey background. CF = Cognitive Functioning, OR = Odds Ratio, CI = Confidence Interval, HN = head-and-neck, D_{mean} = mean dose, $D_{40\%}$ = dose to 40% of the structure volume, Cerebellum_A = anterior cerebellum, Cerebellum_P = posterior cerebellum, Hippocampus_L = left hippocampus, Hippocampus_R = right hippocampus. Bold values represent statistically significant associations.

Variable	**ΔCF 1-year (n=462)**	
	OR (95% CI)	*p*
Primary disease site	n=462	
Brain	[Reference]	
HN	0.81 (0.53 to 1.26)	0.357
Age at RT	n=462	
>65 years	[Reference]	
≤65 years	1.82 (1.24 to 2.65)	**0.002**
Gender	n=462	
Male	[Reference]	
Female	1.10 (0.74 to 1.63)	0.649
Chemotherapy	n=462	
No	[Reference]	
Yes	1.91 (1.29 to 2.83)	**0.001**
Surgery	n=462	
No	[Reference]	
Yes	0.99 (0.67 to 1.46)	0.960
CF Baseline score	n=462	
	1.03 (1.02 to 1.04)	**<0.001**
Brain D_{mean}	n=416	
≤ 1 Gy	[Reference]	
> 1 Gy	1.06 (0.71 to 1.57)	0.773
Brain D_{mean}	n=416	
≤ 3 Gy	[Reference]	
> 3 Gy	1.75 (1.13 to 2.71)	**0.012**
Brain_Supratentorial D_{mean}	n=433	
≤ 1 Gy	[Reference]	
> 1 Gy	1.24 (0.83 to 1.86)	0.291
Brain_Supratentorial D_{mean}	n=433	
≤ 2 Gy	[Reference]	
> 2 Gy	1.52 (0.99 to 2.32)	0.055
Brainstem D_{mean}	n=397	
≤ 2 Gy	[Reference]	
> 2 Gy	1.06 (0.71 to 1.58)	0.789
Brainstem D_{mean}	n=397	
≤ 3 Gy	[Reference]	
> 3 Gy	1.19 (0.79 to 1.79)	0.411
Cerebellum D_{mean}	n=457	
≤ 2 Gy	[Reference]	
> 2 Gy	0.87 (0.60 to 1.27)	0.467
Cerebellum_A D_{mean}	n=396	
≤ 1 Gy	[Reference]	

> 1 Gy	1.08 (0.73 to 1.62)	0.696
Cerebellum_A D_{mean}	n=396	
≤ 3 Gy	[Reference]	
> 3 Gy	1.18 (0.76 to 1.83)	0.470
Cerebellum_P D_{mean}	n=389	
≤ 2 Gy	[Reference]	
> 2 Gy	0.92 (0.62 to 1.38)	0.694
Hippocampus_L D_{mean}	n=179	
≤ 1 Gy	[Reference]	
> 1 Gy	1.88 (1.02 to 3.45)	**0.043**
Hippocampus_L D_{mean}	n=179	
≤ 2 Gy	[Reference]	
> 2 Gy	2.55 (1.36 to 4.75)	**0.003**
Hippocampus_L $D_{40\%}$	n=179	
≤ 1 Gy	[Reference]	
> 1 Gy	1.84 (1.00 to 3.38)	**0.05**
Hippocampus_L $D_{40\%}$	n=179	
≤ 2 Gy	[Reference]	
> 2 Gy	2.40 (1.29 to 4.48)	**0.006**
Hippocampus_R D_{mean}	n=180	
≤ 1 Gy	[Reference]	
> 1 Gy	1.76 (0.96 to 3.23)	0.068
Hippocampus_R D_{mean}	n=180	
≤ 2 Gy	[Reference]	
> 2 Gy	2.43 (1.31 to 4.54)	**0.005**
Hippocampus_R $D_{40\%}$	n=180	
≤ 1 Gy	[Reference]	
> 1 Gy	1.60 (0.87 to 2.93)	0.128
Hippocampus_R $D_{40\%}$	n=180	
≤ 2 Gy	[Reference]	
> 2 Gy	2.30 (1.23 to 4.28)	**0.009**

Table S6 – Univariate logistic regression analysis of the patient characteristics, clinical variables and dose-volume parameters for 1-year ΔCD and ΔEQ6D, for the brain patients only. The number of patients per variable included are given. Δ scores are defined in 2 groups (group 1: improvement or constant, group 2: deterioration). For the dosimetric variables, the limits for dichotomization based on the median DVH values for the neuro patient group (see Table S4) are represented with a grey background. CD = Communication Deficit, OR = Odds Ratio, CI = Confidence Interval, D_{mean} = mean dose, $D_{40\%}$ = dose to 40% of the structure volume, Cerebellum_A = anterior cerebellum, Cerebellum_P = posterior cerebellum, Hippocampus_L = left hippocampus, Hippocampus_R = right hippocampus. Bold values represent statistically significant associations.

**Variable**	**ΔCD 1-year (n=106)**		**ΔEQ6D 1-year (n=105)**	
	OR (95% CI)	*p*	*OR (95% CI)*	*p*
Age at RT	n=106		n=105	
>65 years	[Reference]		[Reference]	
≤65 years	0.73 (0.30 to 1.81)	0.503	0.71 (0.27 to 1.90)	0.498
Gender	n=106		n=105	
Male	[Reference]		[Reference]	
Female	0.89 (0.40 to 1.97)	0.774	1.25 (0.51 to 3.03)	0.626
Chemotherapy	n=106		n=105	
No	[Reference]		[Reference]	
Yes	1.02 (0.45 to 2.28)	0.972	3.77 (1.30 to 10.98)	**0.015**
Surgery	n=106		n=105	
No	[Reference]		[Reference]	
Yes	0.70 (0.27 to 1.79)	0.454	2.76 (0.75 to 10.16)	0.127
CD/EQ6D Baseline score	n=106		n=105	
	0.95 (0.92 to 0.98)	**0.001**	0.56 (0.39 to 1.06)	0.074
Brain D_{mean}	n=104		n=103	
≤ 8 Gy	[Reference]		[Reference]	
> 8 Gy	2.02 (0.90 to 4.53)	0.087	2.90 (1.16 to 7.30)	**0.023**
Brain D_{mean}	n=104		n=103	
≤ 3 Gy	[Reference]		[Reference]	
> 3 Gy	1.00 (0.33 to 3.01)	1	2.82 (0.60 to 13.33)	0.190
Brain_Supratentorial D_{mean}	n=104		n=103	
≤ 8 Gy	[Reference]		[Reference]	
> 8 Gy	1.46 (0.65 to 3.27)	0.360	3.89 (1.41 to 10.71)	**0.009**
Brain_Supratentorial D_{mean}	n=104		n=103	
≤ 2 Gy	[Reference]		[Reference]	
> 2 Gy	2.23 (0.44 to 11.34)	0.333	3.06 (0.36 to 25.67)	0.303
Brainstem D_{mean}	n=99		n=98	
≤ 8 Gy	[Reference]		[Reference]	
> 8 Gy	1.33 (0.59 to 2.99)	0.486	1.17 (0.48 to 2.85)	0.726
Brainstem D_{mean}	n=99		n=98	
≤ 3 Gy	[Reference]		[Reference]	
> 3 Gy	1.24 (0.53 to 2.89)	0.614	1.55 (0.60 to 4.01)	0.370
Cerebellum D_{mean}	n=102		n=101	
≤ 2 Gy	[Reference]		[Reference]	
> 2 Gy	1.09 (0.49 to 2.42)	0.839	1.56 (0.63 to 3.84)	0.334
Cerebellum_A D_{mean}	n=96		n=95	
≤ 6 Gy	[Reference]		[Reference]	
> 6 Gy	1.28 (0.55 to 2.96)	0.564	0.88 (0.36 to 2.18)	0.786
Cerebellum_A D_{mean}	n=96		n=95	
≤ 3 Gy	[Reference]		[Reference]	

> 3 Gy	1.72 (0.72 to 4.13)	0.224	1.16 (0.46 to 2.92)	0.753
Cerebellum_P D_{mean}	n=93		n=92	
≤ 2 Gy	[Reference]		[Reference]	
> 2 Gy	0.96 (0.41 to 2.27)	0.932	0.80 (0.32 to 2.02)	0.632
Cerebellum_P D_{mean}	n=93		n=92	
≤ 3 Gy	[Reference]		[Reference]	
> 3 Gy	0.80 (0.33 to 1.96)	0.632	0.57 (0.21 to 1.54)	0.265
Hippocampus_L D_{mean}	n=101		n=100	
≤ 2 Gy	[Reference]		[Reference]	
> 2 Gy	1.96 (0.84 to 4.56)	0.119	1.74 (0.69 to 4.39)	0.238
Hippocampus_L $D_{40\%}$	n=101		n=100	
≤ 2 Gy	[Reference]		[Reference]	
> 2 Gy	1.73 (0.75 to 3.99)	0.196	1.48 (0.60 to 3.67)	0.395
Hippocampus_R D_{mean}	n=102		n=101	
≤ 3 Gy	[Reference]		[Reference]	
> 3 Gy	1.38 (0.61 to 3.09)	0.441	1.46 (0.60 to 3.55)	0.411
Hippocampus_R $D_{40\%}$	n=102		n=101	
≤ 3 Gy	[Reference]		[Reference]	
> 3 Gy	1.32 (0.59 to 2.94)	0.505	1.08 (0.45 to 2.60)	0.869
Hippocampus_R $D_{40\%}$	n=102		n=101	
≤ 2 Gy	[Reference]		[Reference]	
> 2 Gy	1.35 (0.60 to 3.06)	0.467	1.24 (0.51 to 3.02)	0.642

Table S7 – Univariate logistic regression analysis of the type of chemotherapy for 1-year ΔCF, ΔCD and ΔEQ6D. The number of patients per variable included are given. For the HN patients, only ΔCF is available. Δ scores are defined in 2 groups (group 1: improvement or constant, group 2: deterioration). CD = Communication Deficit, OR = Odds Ratio, CI = Confidence Interval. Bold values represent statistically significant associations.

**Type of chemotherapy**	**ΔCF 1-year (n=107)**		**ΔCD 1-year**		**ΔEQ6D 1-year**	
	OR (95% CI)	*p*	*OR (95% CI)*	*p*	*OR (95% CI)*	*p*
Brain patients	n=107		n=106		n=105	
PCV (n=14)	0.49 (0.14 to 1.66)	0.250	0.11 (0.01 to 0.87)	**0.037**	0.44 (0.09 to 2.11)	0.304
TMZ (n=53)	2.19 (1.00 to 4.78)	**0.049**	2.00 (0.90 to 4.46)	0.090	4.57 (1.73 to 12.11)	**0.002**
HN patients	n=355					
Cisplatin (n=76)	1.88 (1.12 to 3.13)	**0.016**				
Carboplatin (n=2)	-	-				
Cetuximab (n=17)	1.48 (0.56 to 3.93)	0.434				

Table S8 – Multivariate logistic regression analysis of patient characteristics, clinical variables and dose-volume parameters for 1-year ΔCD and ΔEQ6D, for the brain patients only. Δ scores are defined in 2 groups (group 1: improvement or constant, group 2: deterioration). CD = Communication Deficit, OR = Odds Ratio, CI = Confidence Interval, D_{mean} = mean dose, Cerebellum_A = anterior cerebellum, Cerebellum_P = posterior cerebellum. Bold values represent statistically significant associations.

	**ΔCD 1-year**		**ΔEQ6D 1-year**	
	**OR (95% CI)**	**p**	**OR (95% CI)**	**p**
**Patient characteristics model**	n=106		n=105	
Age at start RT ≤65 years	0.77 (0.28 to 2.12)	0.612	0.44 (0.14 to 1.39)	0.162
Gender = female	0.70 (0.29 to 1.68)	0.423	1.31 (0.50 to 3.41)	0.585
Chemotherapy = yes	1.07 (0.42 to 2.72)	0.892	4.31 (1.31 to 14.14)	**0.016**
Surgery = yes	0.89 (0.31 to 2.55)	0.823	2.41 (0.59 to 9.89)	0.223
CD Baseline score	0.95 (0.92 to 0.98)	**0.001**	0.55 (0.28 to 1.10)	0.093
**Dose-volume measures model 1 (median DVH values)**	n=88		n=87	
Brain D_{mean} (> 8 Gy vs. ≤ 8 Gy)	2.24 (0.90 to 5.57)	0.083	2.52 (0.94 to 6.73)	0.065
Brainstem D_{mean} (> 8 Gy vs. ≤ 8 Gy)	0.91 (0.20 to 4.03)	0.897	0.87 (0.18 to 4.23)	0.863
Cerebellum_A D_{mean} (> 6 Gy vs. ≤ 6 Gy)	1.54 (0.20 to 11.61)	0.675	1.02 (0.12 to 8.73)	0.989
Cerebellum_P D_{mean} (> 2 Gy vs. ≤ 2 Gy)	0.72 (0.15 to 3.48)	0.680	0.84 (0.15 to 4.55)	0.836
**Dose-volume measures model 2 (most significant DVH values)**	n=88		n=87	
Brain D_{mean} (> 3 Gy vs. ≤ 3 Gy)	1.66 (0.41 to 6.65)	0.477	3.05 (0.57 to 16.28)	0.192
Brainstem D_{mean} (> 3 Gy vs. ≤ 3 Gy)	0.37 (0.06 to 2.17)	0.269	1.41 (0.26 to 7.57)	0.689
Cerebellum_A D_{mean} (> 3 Gy vs. ≤ 3 Gy)	5.68 (0.82 to 39.42)	0.079	1.60 (0.27 to 9.29)	0.604
Cerebellum_P D_{mean} (> 3 Gy vs. ≤ 3 Gy)	0.47 (0.15 to 1.50)	0.204	0.36 (0.10 to 1.24)	0.104
**Combined model 1 (median DVH values)**	n=88		n=87	
Age at start RT ≤65 years	1.06 (0.32 to 3.53)	0.922	0.47 (0.14 to 1.61)	0.232
Gender = female	0.79 (0.27 to 2.33)	0.665	1.21 (0.43 to 3.40)	0.719
Chemotherapy = yes	0.75 (0.15 to 3.74)	0.722	2.65 (0.56 to 12.67)	0.221
Surgery = yes	0.79 (0.21 to 2.99)	0.731	2.38 (0.51 to 11.11)	0.271
CD Baseline score	0.93 (0.89 to 0.97)	**<0.001**	0.52 (0.25 to 1.09)	0.082
Brain D_{mean} (> 8 Gy vs. ≤ 8 Gy)	2.86 (0.67 to 12.15)	0.155	1.28 (0.35 to 4.69)	0.711
Brainstem D_{mean} (> 8 Gy vs. ≤ 8 Gy)	1.23 (0.24 to 6.38)	0.802	1.22 (0.22 to 6.73)	0.821
Cerebellum_A D_{mean} (> 6 Gy vs. ≤ 6 Gy)	1.84 (0.18 to 18.88)	0.609	1.31 (0.12 to 14.20)	0.825
Cerebellum_P D_{mean} (> 2 Gy vs. ≤ 2 Gy)	0.60 (0.09 to 3.95)	0.598	0.59 (0.09 to 4.04)	0.589
**Combined model 2 (most significant DVH values)**	n=88		n=87	
Age at start RT ≤65 years	0.82 (0.24 to 2.79)	0.752	0.37 (0.10 to 1.350)	0.132
Gender = female	0.92 (0.30 to 2.81)	0.888	1.19 (0.40 to 3.52)	0.755
Chemotherapy = yes	1.47 (0.39 to 5.50)	0.567	3.03 (0.72 to 12.72)	0.129
Surgery = yes	0.70 (0.18 to 2.82)	0.617	2.37 (0.46 to 12.13)	0.301
CD Baseline score	0.93 (0.89 to 0.97)	**<0.001**	0.50 (0.23 to 1.05)	0.068
Brain D_{mean} (> 3 Gy vs. ≤ 3 Gy)	3.25 (0.50 to 20.88)	0.215	1.66 (0.21 to 13.04)	0.631
Brainstem D_{mean} (> 3 Gy vs. ≤ 3 Gy)	0.44 (0.06 to 3.23)	0.416	1.11 (0.17 to 7.25)	0.916
Cerebellum_A D_{mean} (> 3 Gy vs. ≤ 3 Gy)	7.58 (0.78 to 73.46)	0.080	2.57 (0.35 to 18.82)	0.354
Cerebellum_P D_{mean} (> 3 Gy vs. ≤ 3 Gy)	0.37 (0.09 to 1.52)	0.167	0.27 (0.06 to 1.13)	0.073

Table S9 – Regression weight coefficients of the multivariate logistic regression models for ΔCF as specified in the manuscript. Given are the variables included in the model as well as the constant. The standard error is reported in brackets. CF = Cognitive Functioning, CD = Communication Deficit, D_{mean} = mean dose, Cerebellum_A = anterior cerebellum, Cerebellum_P = posterior cerebellum.

	**ΔCF 1-year**
**Patient characteristics model**	
Primary disease site = HN	-0.28 (0.30)
Age at start RT ≤65 years	0.55 (0.23)
Gender = female	0.08 (0.23)
Chemotherapy = yes	0.46 (0.24)
Surgery = yes	-0.10 (0.25)
CF Baseline score	0.03 (0.01)
Constant	-3.47 (0.61)
**Dose-volume measures model 1 (median DVH values)**	
Brain D_{mean} (> 1 Gy vs. ≤ 1 Gy)	0.00 (0.28)
Brainstem D_{mean} (> 2 Gy vs. ≤ 2 Gy)	0.11 (0.42)
Cerebellum_A D_{mean} (> 1 Gy vs. ≤ 1 Gy)	0.26 (0.43)
Cerebellum_P D_{mean} (> 2 Gy vs. ≤ 2 Gy)	-0.22 (0.31)
Constant	-0.45 (0.19)
**Dose-volume measures model 2 (most significant DVH values)**	
Brain D_{mean} (> 3 Gy vs. ≤ 3 Gy)	0.71 (0.29)
Brainstem D_{mean} (> 3 Gy vs. ≤ 3 Gy)	-0.01 (0.35)
Cerebellum_A D_{mean} (> 3 Gy vs. ≤ 3 Gy)	-0.08 (0.37)
Cerebellum_P D_{mean} (> 2 Gy vs. ≤ 2 Gy)	0.06 (0.27)
Constant	-0.60 (0.19)
**Combined model 1 (median DVH values)**	
Primary disease site = HN	-0.21 (0.41)
Age at start RT ≤65 years	0.51 (0.27)
Gender = female	-0.05 (0.26)
Chemotherapy = yes	0.53 (0.27)
Surgery = yes	-0.08 (0.28)
CF Baseline score	0.04 (0.01)
Brain D_{mean} (> 1 Gy vs. ≤ 1 Gy)	-0.09 (0.37)
Brainstem D_{mean} (> 2 Gy vs. ≤ 2 Gy)	0.42 (0.44)
Cerebellum_A D_{mean} (> 1 Gy vs. ≤ 1 Gy)	0.15 (0.45)
Cerebellum_P D_{mean} (> 2 Gy vs. ≤ 2 Gy)	-0.25 (0.35)
Constant	-3.87 (0.77)
**Combined model 2 (most significant DVH values**	
Primary disease site = HN	0.67 (0.47)
Age at start RT ≤65 years	0.58 (0.27)
Gender = female	-0.02 (0.26)
Chemotherapy = yes	0.42 (0.28)
Surgery = yes	-0.21 (0.29)
CF Baseline score	0.04 (0.01)
Brain D_{mean} (> 3 Gy vs. ≤ 3 Gy)	1.20 (0.46)
Brainstem D_{mean} (> 3 Gy vs. ≤ 3 Gy)	0.13 (0.37)

Cerebellum_A D_{mean} (> 3 Gy vs. ≤ 3 Gy)	0.16 (0.41)
Cerebellum_P D_{mean} (> 2 Gy vs. ≤ 2 Gy)	-0.15 (0.30)
Constant	-4.74 (0.82)

Table S10 – Regression weight coefficients of the multivariate logistic regression models for ΔCD and ΔEQ6D as specified in the manuscript. Given are the variables included in the model as well as the constant. The standard error is reported in brackets. CD = Communication Deficit, D_{mean} = mean dose, Cerebellum_A = anterior cerebellum, Cerebellum_P = posterior cerebellum.

	**ΔCD 1-year**	**ΔEQ6D 1-year**
**Patient characteristics model**		
Age at start RT ≤65 years	-0.26 (0.52)	-0.82 (0.59)
Gender = female	-0.36 (0.45)	0.27 (0.49)
Chemotherapy = yes	0.07 (0.48)	1.46 (0.61)
Surgery = yes	-0.12 (0.54)	0.88 (0.72)
CD/EQ6D Baseline score	-0.05 (0.02)	-0.59 (0.35)
Constant	0.55 (0.63)	-1.31 (0.95)
**Dose-volume measures model 1 (median DVH values)**		
Brain D_{mean} (> 8 Gy vs. ≤ 8 Gy)	0.81 (0.47)	0.92 (0.50)
Brainstem D_{mean} (> 8 Gy vs. ≤ 8 Gy)	-0.10 (0.76)	-0.14 (0.81)
Cerebellum_A D_{mean} (> 6 Gy vs. ≤ 6 Gy)	0.43 (1.03)	0.02 (1.10)
Cerebellum_P D_{mean} (> 2 Gy vs. ≤ 2 Gy)	-0.33 (0.81)	-0.18 (0.87)
Constant	-0.97 (0.39)	-1.23 (0.42)
**Dose-volume measures model 2 (most significant DVH values)**		
Brain D_{mean} (> 3 Gy vs. ≤ 3 Gy)	0.51 (0.71)	1.12 (0.86)
Brainstem D_{mean} (> 3 Gy vs. ≤ 3 Gy)	-1.00 (0.91)	0.34 (0.86)
Cerebellum_A D_{mean} (> 3 Gy vs. ≤ 3 Gy)	1.74 (0.99)	0.47 (0.90)
Cerebellum_P D_{mean} (> 3 Gy vs. ≤ 3 Gy)	-0.75 (0.59)	-1.03 (0.63)
Constant	-1.09 (0.72)	-2.01 (0.89)
**Combined model 1 (median DVH values)**		
Age at start RT ≤65 years	-0.20 (0.62)	-0.99 (0.66)
Gender = female	-0.08 (0.57)	0.17 (0.55)
Chemotherapy = yes	0.39 (0.67)	1.11 (0.73)
Surgery = yes	-0.35 (0.71)	0.86 (0.83)
CD/EQ6D Baseline score	-0.08 (0.02)	-0.70 (0.38)
Brain D_{mean} (> 3 Gy vs. ≤ 3 Gy)	1.18 (0.95)	0.51 (1.05)
Brainstem D_{mean} (> 3 Gy vs. ≤ 3 Gy)	-0.83 (1.02)	0.10 (0.96)
Cerebellum_A D_{mean} (> 3 Gy vs. ≤ 3 Gy)	2.03 (1.16)	0.94 (1.02)
Cerebellum_P D_{mean} (> 3 Gy vs. ≤ 3 Gy)	-1.01 (0.73)	-1.32 (0.74)
Constant	-0.73 (1.03)	-1.17 (1.18)
**Combined model 2 (most significant DVH values)**		
Age at start RT ≤65 years	0.06 (0.61)	-0.75 (0.63)
Gender = female	-0.24 (0.56)	0.19 (0.53)
Chemotherapy = yes	-0.29 (0.82)	0.98 (0.80)
Surgery = yes	-0.23 (0.68)	0.87 (0.79)
CD/EQ6D Baseline score	-0.08 (0.02)	-0.65 (0.37)
Brain D_{mean} (> 8 Gy vs. ≤ 8 Gy)	1.05 (0.74)	0.25 (0.66)

Brainstem D_{mean} (> 8 Gy vs. ≤ 8 Gy)	0.21 (0.84)	0.20 (0.87)
Cerebellum_A D_{mean} (> 6 Gy vs. ≤ 6 Gy)	0.61 (1.19)	0.27 (1.22)
Cerebellum_P D_{mean} (> 2 Gy vs. ≤ 2 Gy)	-0.51 (0.96)	-0.53 (0.98)
Constant	0.12 (0.81)	-0.84 (1.02)
